# Minimally Invasive Micro-Indentation: mapping tissue mechanics at the tip of an 18G needle

**DOI:** 10.1038/s41598-017-10526-4

**Published:** 2017-09-12

**Authors:** Steven V. Beekmans, Kaj S. Emanuel, Theodoor H. Smit, Davide Iannuzzi

**Affiliations:** 1 Department of Physics and Astronomy and LaserLab Amsterdam, Vrije Universiteit Amsterdam, De Boelelaan 1085, 1081 HV Amsterdam, Netherlands; 2 Department of Orthopaedic Surgery, VU University Medical Center (VUmc), Amsterdam Movement Sciences, De Boelelaan 1117, 1081 HV Amsterdam, Netherlands; 3 Department of Medical Biology and Department of Orthopedic Surgery, Academic Medical Center (AMC), Meiberdreef 9, 1105 AZ Amsterdam, Netherlands

## Abstract

Experiments regarding the mechanical properties of soft tissues mostly rely on data collected on specimens that are extracted from their native environment. During the extraction and in the time period between the extraction and the completion of the measurements, however, the specimen may undergo structural changes which could generate unwanted artifacts. To further investigate the role of mechanics in physiology and possibly use it in clinical practices, it is thus of paramount importance to develop instruments that could measure the viscoelastic response of a tissue without necessarily excising it. Tantalized by this opportunity, we have designed a minimally invasive micro-indenter that is able to probe the mechanical response of soft tissues, *in situ*, via an 18G needle. Here, we discuss its working principle and validate its usability by mapping the viscoelastic properties of a complex, confined sample, namely, the nucleus pulposus of the intervertebral disc. Our findings show that the mechanical properties of a biological tissue in its local environment may be indeed different than those that one would measure after excision, and thus confirm that, to better understand the role of mechanics in life sciences, one should always perform minimally invasive measurements like those that we have here introduced.

## Introduction

It is widely recognized that the micromechanical environment that surrounds cells and biological tissues can influence a large number of fundamental physiological processes, including cell growth, cell signaling, cell migration, tumor development, angiogenesis, wound healing, scar formation, and even a highly complex process such as stem cell differentiation^1–8^. In the field of tissue engineering, it has been further demonstrated that the mechanical stability at the defect site of the host is of key importance for the growth of successful biocompatible materials^9,10^. It is thus not surprising that, over the last decade, there has been increasing attention to the development of experimental tools and methods that could locally map the mechanical properties of cells and tissues for both fundamental research and clinical applications^11–16^.

At present, the characterization of the mechanical properties of biological samples is still mostly carried out by means of (micro-)indentation^17^. For instance, recent indentation experiments on brain tissue–one of the softest tissues of the human body–have proven to offer valuable insights in the mechanics behind the structural heterogeneity that forms the gray and white matter. These studies hold promise for a better understanding of the response of brain tissue to life threatening conditions as severe as cancerous tumors, Alzheimer’s, and traumatic injury^18–20^. On the other side of the scale, in the field of orthopedics, indentation is known to provide previously neglected mechanical information on a tissue as stiff as cartilage^21^, proving the great versatility of the approach.

(Micro-)indentation experiments, however, suffer from one major limitation. To perform the measurement of the mechanical properties of a sample, the head of the (micro-)indenter, which is typically rather bulky, needs to move into contact with the top surface. Experiments can thus be performed only on excised specimens. Yet, the extraction and preservation protocol can introduce biases that may lead to wrong conclusions. The mechanical properties of most sensitive tissues, in fact, can be altered by drying, swelling and loss of confinement, or external stress^22–24^. One can conclude that an unbiased recording of tissue mechanical properties can only be obtained when the target tissue is still within the original surroundings. There is thus a strong demand for a device that could measure the mechanics of a sample below its surface via non- or minimally invasive means, and, in that way, enable true *in situ* characterization of tissue viscoelasticity^25–27^. In 2006, for example, Imer and colleagues introduced the so-called *scanning force arthroscope*, which is able to perform *in vivo* nano-indentation measurements during a standard arthroscopic procedure. Unfortunately, this instrument relies on a bulky stabilization module that prevents minimally invasive experiments^28^. More recently, our group has developed a needle-based micro-indenter, which, however, can only operate via apertures as large as 5 millimeters^26^. Alternative non-invasive techniques, such as optical coherence elastography, magnetic resonance elastography, and ultrasound elastography, certainly circumvent the excision issue, but are limited to provide the mechanical properties of tissues only at a relatively macroscopic scale and generally lack the ability to provide quantitative results^27,29,30^.

To solve this impasse, in this paper, we present a new micro-indenter that is able to probe the heterogeneous viscoelastic properties of soft tissues *in situ* through an 18G needle. The indenter is based on ferrule-top technology and employs the bending of a micro-machined cantilever to infer the viscoelastic properties of a sample by means of dynamic mechanical analysis (DMA)^31–33^. We validate the working principle of our Minimally Invasive Micro-Indentation (MIMI) on a silicon polymer that is often used as a substitute for soft tissues. To demonstrate the full potential of the approach proposed, we further present a dynamic mechanical analysis of the nucleus pulposus (NP) of a goat’s intervertebral disc (IVD) obtained by inserting the needle into the annulus fibrosus (AF), i.e., without extracting the NP from the IVD.

## Results

**Figure 1:**
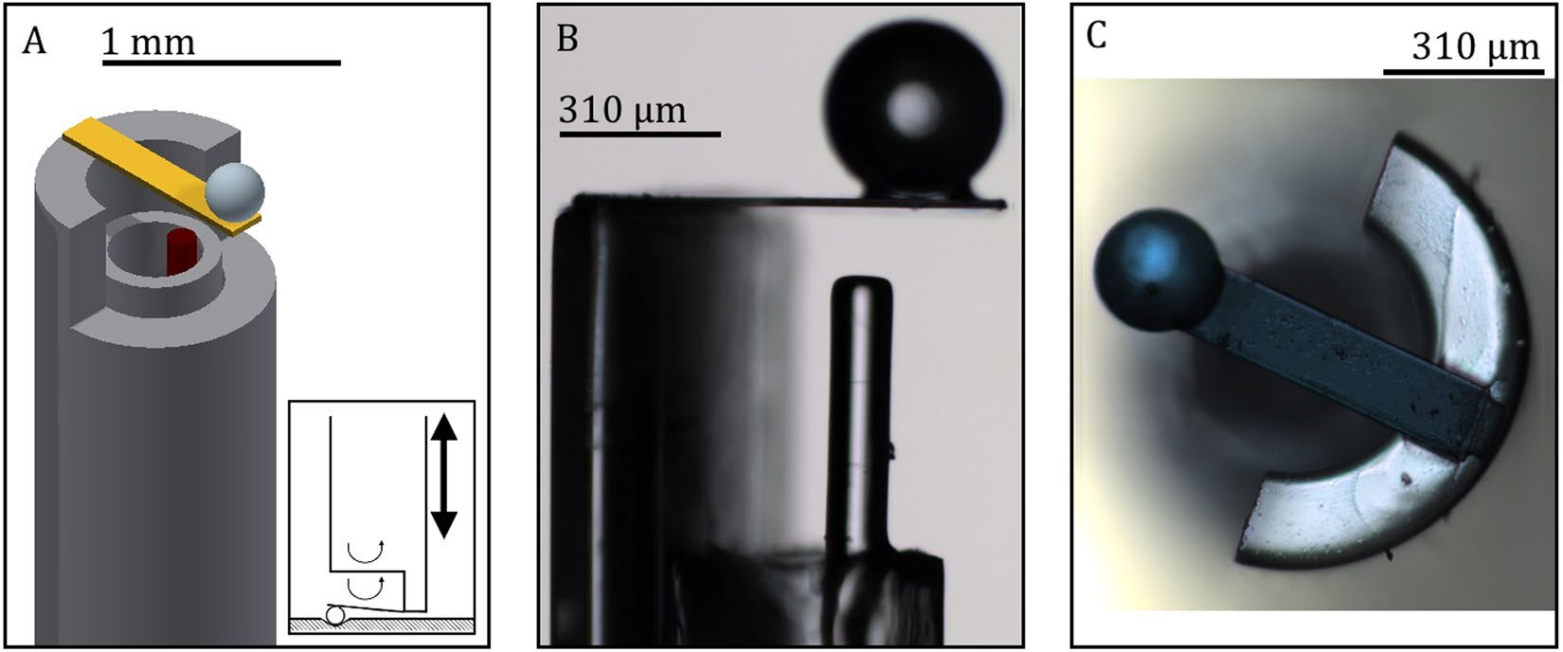
Sketches and microscope images of the optical force transducer used at the tip of the MIMI indenter. (**A**) Schematic model of the probe, which consists of a cantilever indentation spring (gold), an optical fiber for the interferometric readout of the displacement of the cantilever (red), and a borosilicate sphere to create a spherical contact with the indented surface (blue). The inset shows a schematic of the indentation procedure, where emphasis is put on the interferometric readout and the movement of the piezoelectric transducer (not-to-scale); (**B**) Microscope image of the probe, showing the interferometric cavity; (**C**) Top view of the sensor.

The main purpose of this study was to design a minimally invasive *in situ* indenter for viscoelastic characterization of tissues below the top surface. The indenter is based on a micro-machined cantilever spring operating as force transducer, the displacement of which is monitored by a Fabry-Pérot interferometer. The free hanging end of the cantilever is equipped with a spherical tip, which is used to indent the tissue (Fig. 1). After needle insertion, a piezoelectric manipulator, fixed at the proximal end of the needle, advances the probe inside the needle until a predefined load on the sample is achieved. After contact has been reached, a sinusoidal frequency sweep is imposed on the cantilever and the indentation response of the sample is recorded to determine the frequency dependent storage- and loss moduli of the sample. The fabrication details of the probe and the measurement protocol are further discussed in the Methods section (see also ref.^31^).

**Figure 2:**
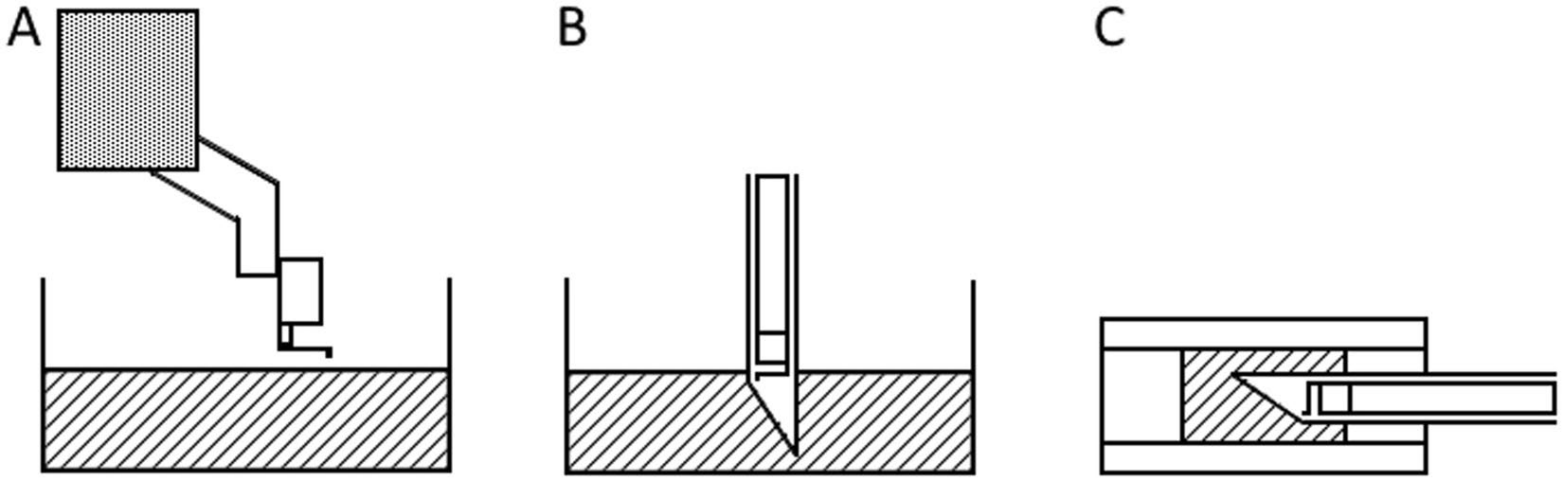
Schematic overview of the measurements performed to validate the MIMI indenter. The areas shaded with diagonal lines represent the PDMS sample. (**A**) First experiment: indentation of the top surface of a PDMS sample performed with a validated table top indenter as presented in ref.^31^. (**B**) Second experiment: indentation inside a PDMS sample performed with our MIMI indenter. (**C**) Third experiment: indentation inside a PDMS sample inside the NP of an IVD performed with our MIMI indenter. Not-to-scale.

To validate our instrument against an established technique, we measured the storage and loss moduli of a polymeric reference sample (Poly(DiMethyl)Siloxane (PDMS) with a crosslinker:prepolymer weight ratio of 1:20) in three different experiments. The first experiment (Fig. 2A) was performed on the top surface of the sample by means of a table top indenter described in previous research, which was thoroughly validated against traditional macroscopic shear rheology^31^. In the second experiment (Fig. 2B), we used our MIMI indenter to pierce the same sample and measure the mechanical properties of the material in the bulk. For the third experiment (Fig. 2C), which is designed to test for any systematic errors of our instrument after insertion through a rigid material, we prepared a dummy sample obtained by replacing the NP of a goat’s IVD with a polymeric sample identical to the one used in the previous two experiments. Measurements of the mechanical properties of the dummy NP were then carried out by inserting our MIMI indenter through the AF. For more details, we refer the reader to the Methods section.

**Figure 3:**
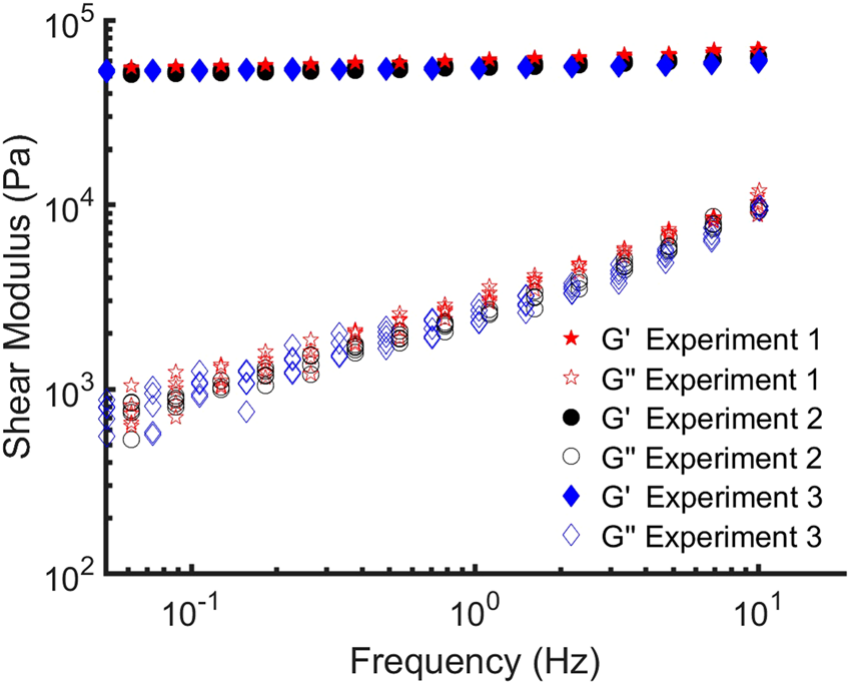
Quantitative comparison of the storage and loss moduli obtained in the three experiments sketched in Fig. 2, plotted as a function of the probed frequency.

Figure 3 shows the dynamic response of the PDMS for a frequency range of 0.05–10 Hz as measured in the three experiments. Each frequency scan (containing 15 frequencies) was fully independent and no fitting was applied. Storage and loss moduli (*G*′ and *G*′′, respectively) are presented for 5 frequency sweeps per experiment. All five sweeps were performed in the same location. It can be observed from Fig. 3 that the frequency dependent storage and loss moduli are in quantitative agreement with each other.

To demonstrate the capabilities of the MIMI indenter in full, we have further measured the storage and loss moduli of the NP without extracting it from the IVD.

**Figure 4:**
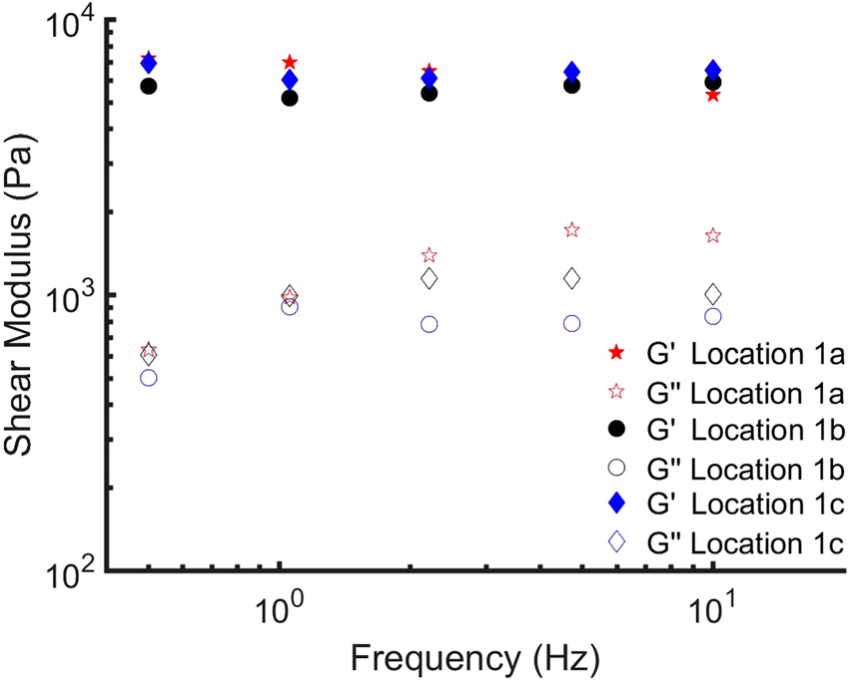
Dynamic response of the NP of disc 1, for a frequency range of 0.5–10 Hz, measured *in situ* using the needle-based MIMI probe. We performed indentations on three independent locations. Each location represents an average of 5 frequency sweeps. Variability of *G*′ (closed symbols) and *G*′′ (open symbols) is found to be marginal within one disc.

**Figure 5:**
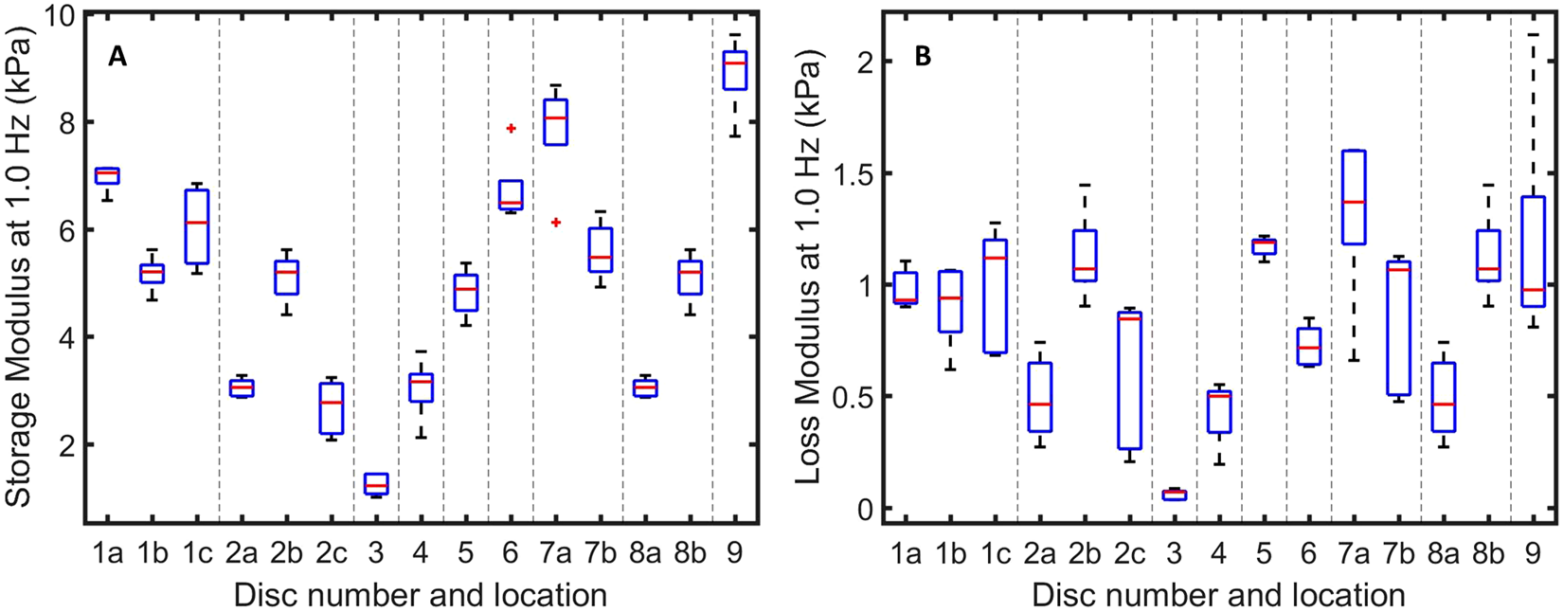
Boxplot of the *in situ* storage- (**A**) and loss (**B**) modulus of the NP at 1.0 Hz. Entries are separated by disc. Locations within a disc are indicated by letters. Even though a large variety in *G*′ and *G*′′ is observed between discs, the variation between different locations within one disc is minor.

The NP is a proteoglycan-rich type of connective tissue confined between the two endplates of the disc and the AF. It is known to play a crucial role in the mechanical function of the IVD and its degeneration is considered one of the underlying factors of low back pain^34,35^. An accurate assessment of the mechanical properties of this tissue is thus extremely relevant to a better understanding of the causes of certain conditions and on the definition of protocols for engineered replacement materials. The mechanical properties of the NP, however, may be drastically altered when the original confinement of the NP is released or when brought in contact with air or a liquid with unphysiological osmolarity. Not surprisingly, different rheology measurements on extracted NPs, performed according to various protocols, do provide different values for the elastic and viscous moduli of the material^36–40^. Our needle-based indenter, however, gives us the opportunity to record the *in situ* values of *G*′ and *G*′′ of the NP while maintaining the original confinement and environment inside the disc. In our measurements, we decided to limit the frequency of the sweep to 0.5–10 Hz, as measuring at faster timescales reduces the influence of time dependent changes that occur in soft, hydrated tissues. Moreover, for the sake of measurement time, we reduced the number of frequencies in the sweep to 5. A longer measurement time would lead to increased deterioration in the sample, which may cause a change in viscoelastic properties over time. In Fig. 4 we report the results obtained by probing three different locations within the same IVD. The moduli reported for each location are averaged over 5 frequency scans. Figure 5 further shows the observed *in situ* storage and loss modulus at 1.0 Hz on 9 different IVD. Each box represents five measurements taken in the same location.

## Discussion

The agreement between the results of the first and second experiment on the PDMS sample demonstrates that our MIMI approach is able to accurately capture the viscoelastic properties of an homogeneous material below its top surface. The agreement of these two sets of data with those obtained with the dummy IVD further shows that the insertion of the needle through a rigid material (i.e., the AF) does not introduce any systematic error in the measurements. Moreover, the decreasing trend of the loss modulus of PDMS with decreasing frequency is well-known and confirmed by literature^41^.

As for the experiments performed on the IVD, data collected on the same disc were found to be consistent. Measurements performed on different discs, however, seem to suggest that the mechanical properties of the NP vary significantly from sample to sample, as already reported in the literature^36–38^. The storage modulus of the NP varied between 1.7 and 7.2 kPa over all the recorded discs. These quantitative differences in *G*′ may be described to the position of the disc in the spine of the animal^39,42^.

Most importantly, we have observed a sharp contrast between the NP moduli for goat IVD measured *in situ* and those reported in the literature after extraction, which were presented in the order of 10–50 kPa^38^. This order of magnitude difference may be caused by the extensive procedure that is required to extract the NP from the IVD. Prior to rheological testing, the IVD is cut open and the nucleus is surgically removed, both of which are procedures that could damage NP structure. Moreover, during sample preparation the NP is exposed to air and the original confinement is lost, resulting in drying and swelling. The loss of confinement and the resulting swelling of the nucleus tissue may lead to the higher storage modulus reported in the literature. The NP, in fact, consists of large amounts water-binding proteoglycans, embedded in loosely structured collagen fibrils, which are not fully stretched in their native state due to the confinement conditions^35^. Upon release of these constraints, we hypothesize that the swelling enables the collagen fibers to fully stretch, thereby increasing the stiffness of the tissue.

From the results obtained in the IVD experiments, we conclude that the mechanical properties of the NP can be accurately observed only when the NP is probed in its native environment, where the tissue is properly confined and exposed to its natural hydration conditions. One can further extrapolate this result to the numerous other tissues that, when inside the human body, are exposed to strain (such as skin, arteries, veins), confinement (brain, eye), or swelling. These highly sensitive tissues may all have a strong mechanical reaction to extraction from their surroundings. Measurements *in situ* are therefore mandatory for a quantitative assessment of the role of mechanics in their development or degradation. Similarly, we propose that biomaterials designed for integration in the body should be mechanically characterized in an environment that resembles their intended area–such as a loaded disc culture system^43^ – to avoid a possible mismatch between *in situ* and *ex situ* tissue mechanical properties.

In conclusion, we have introduced an indenter that can perform accurate measurements of the elastic and viscous properties of a material at the end of an 18G needle. We believe that the localized and minimally invasive character of MIMI measurements, combined with the versatility of the probe it is based on, may soon trigger an entire new generation of experiments that will enable a deeper understanding of the role of mechanics in physiology and tissue engineering.

## Outlook and Limitations

We have chosen to limit the band of the frequency sweep to 0.05–10 Hz, corresponding to timescales in which most natural processes occur. Oscillations with very low frequencies (*F* < 0.05 Hz) may be biologically significant but were not attainable for *in situ* application. The analysis of *G*′′ is highly sensitive to perturbations in the low frequency range, as the energy stored in the sample (and therefore the obtained phase shift) at those frequencies is minimal. Friction in the needle shaft caused by piercing the AF tissue hampered the movement of the probe in the lumen of the needle when the speed was very low, resulting in an erroneous phase estimation of the oscillating indentation signal. The upper limit of the frequency band is dependent on the resonance frequency of the piezoelectric translator and can be increased when a translator with a smaller maximum displacement is selected.

One of the main limitations of our force sensor is the fragility of the cantilever. During the course of this study we had to replace the sensor several times due to failure as a result of overloading or obstruction of the cantilever. Future experiments may benefit from force sensors with innovative designs such as membranes or MEMS based structures. Miniaturization of the sensor to sub-millimeter size would reduce the impact on the sample even more.

## Methods

### Force transducer and readout

**Figure 6:**
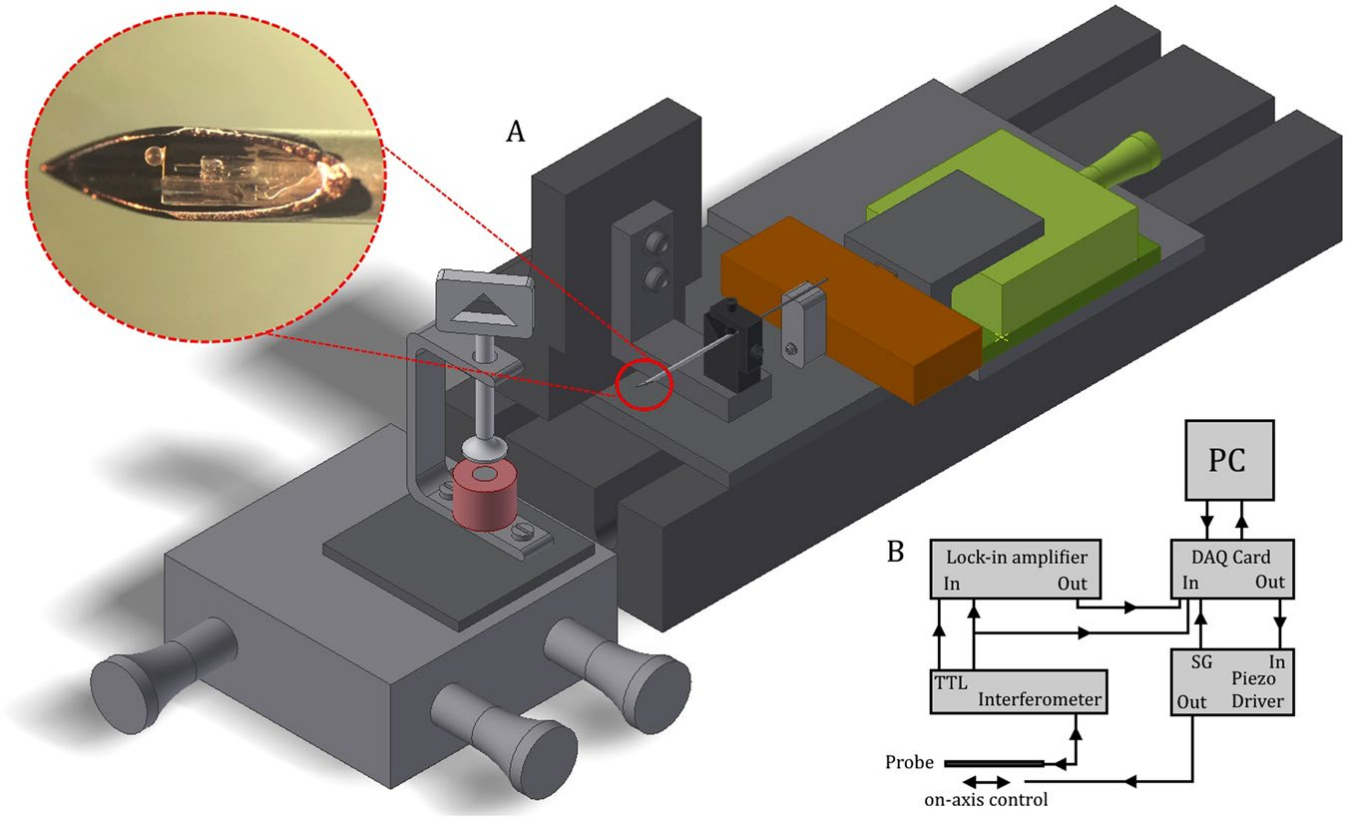
Schematic of the (**A**) experimental setup and (**B**) readout and feedback control. (**A**) The probe is mounted on a long-range piezoelectric translator (orange), which is attached to a manual translation stage (green). The probe is housed in an 18G needle, fixed on a motorized linear stage. The sample is clamped in front of the needle and can be positioned in three dimensions (XYZ). (**B**) Real-time control of cantilever displacement (i.e. load control) by means of the piezoelectric translator is enabled by high-frequency wavelength modulation.

The optical force transducer was built in-house out of borosilicate parts and consisted of a cantilever mounted on top of a cleaved single mode optical fiber. An extensive description of probe fabrication can be found elsewhere, although probes have been slightly adapted for this study^31^. Instead of using a ferrule, we mounted the cantilever on an 8 cm long borosilicate capillary (diameter: 1 mm, wall thickness: 0.21 mm, Science Products GmbH), as illustrated in Fig. 1. The optical fiber (Corning SMF-28) is supported by a second borosilicate capillary (diameter: 0.55 mm, wall thickness: 0.075 mm, Vitrocom), rigidly mounted inside the first capillary. A Fabry-Pérot cavity was created between the cleaved facet of the fiber and the cantilever by coupling the distal end of the fiber to an interferometer (OP1550 V2, Optics11). The recorded intensity signal on the detector encodes for the deflection of the cantilever, which can be obtained by lock-in detection, as described in previous work^44^. A schematic of the experimental setup is presented in Fig. 6. The probe was mounted on a long-range piezoelectric transducer (P-602.5L8, Physik Instrumente GmbH), which in turn was mounted on a coarse positioning stage. To enable a minimally invasive measurement, the probe was inserted into an 18G needle (in-house fabricated from a stainless steel capillary with diameter: 1.3 mm, wall thickness: 0.1 mm, Salomon’s metalen b.v.) and was able to move freely in axial direction with respect to the needle thanks to the piezoelectric transducer and a manipulator. The needle was fixed on a motorized linear stage (LTS300, Thorlabs GmbH), which was used for needle insertion.

Before installation in the needle, the spring constant of newly fabricated probes was calibrated using an in-house developed calibration method^44^. Cantilever spring constants varied between 60–70 N/m, slightly depending on the exact position of the spherical indentation tip at the far end of the cantilever. Prior to each experiment, adequate probe performance was confirmed by a calibration procedure on glass. Whenever a probe did not perform satisfactory due to drifts or bad interference fringe visibility, it was discarded and replaced. A possible geometrical offset between the position of the cleaved optical fiber and that of the spherical tip was accounted for during the calibration procedure on glass.

### Dynamic mechanical analysis

DMA can be used to obtain information about the dynamic mechanical moduli of a sample. Its application to indentation has been described in previous research^31,33^. By means of a feedback control loop on the bending of the cantilever a predefined oscillatory load-sweep was applied on top of a fixed static load. Control of the applied load (i.e. cantilever deflection, instead of only probe movement) was essential to ensure that a consistent stress was applied to the sample at each indentation. Upon contact with the tissue, the feedback-controlled piezoelectric transducer moved the probe forward (thus bending the cantilever) until a predefined value for the applied load (~300) by the cantilever was reached. This load was kept stable for at least 60s to allow for dissipation of the tissue. Afterwards, the load was oscillated sequentially (amplitude ~10) for a finite number of increasing frequencies (5 periods each), logarithmically spaced between 0.05 Hz (0.5 Hz for the NP measurements) and 10 Hz. During all indentations it was ensured that the indentation depth stayed within the linear viscoelastic regime and that indentation depth was much smaller than the bead radius (the maximum static indentation depth was 40). Shear storage and loss moduli were obtained by employing an analytical solution for oscillatory indentation using a spherical indenter obtained in previous research^31,33^.

Although our indenter is limited to a compressive motion, moduli in this study are presented as shear moduli (i.e. the ratio of shear stress to shear strain). The modulus of compression (*E*) was converted into the shear modulus (*G*) by means of Poission’s ratio, which was assumed to be 0.5 for roughly incompressible soft biological tissue^45,46^. We have explicitly made the conversion to shear modulus to facilitate a quantitative comparison of our *in situ* results with classical rheology as well as other shear-based techniques.

### Measurement protocol

During the *in situ* measurements discs were placed in a clamp mounted on top of a three-axis micro manipulation stage (MAX312D, Thorlabs GmbH) and positioned in front of the needle (Fig. 6). Prior to the first insertion, discs were probed with a 21G hypodermic needle (Neolus NN-2138R, Terumo) to locate a suitable insertion trajectory. Subsequently, the 18G needle, housing the micro-indenter, was inserted through the annulus. All measurements were performed 1–3 mm inside the NP. After reaching the target location, insertion of the needle was stopped and the probe was carefully advanced until contact with the tissue was observed. In case of contamination of the lumen of the needle, which was indicated by premature contact with the tissue, the probe and the needle were retracted and the lumen was cleaned. After finding contact with the tissue, the probe was retracted for 100 and the dynamic mechanical analysis procedure was started (see section Dynamic mechanical analysis). Five frequency sweeps were performed for each needle insertion. After indentation, the probe and needle were retracted to their respective stating positions.

For the *ex situ* reference measurements the probe was positioned such that it slightly protruded the tip of the needle. The sample was placed vertically in front of the device and the probe was moved forward until contact was found. Afterwards, the aforementioned measurement procedure was followed.

### Intervertebral disc preparation

Isolated intact spines of skeletally mature female milk goats (age 3–4 years) were obtained from a local butcher and processed immediately after isolation. To prepare specimens for testing, individual discs were separated from the spine with an oscillating saw, maintaining 2–5 mm of endplate on both sides. Subsequently, discs were brushed clean, rinsed and stored in physiological saline soaked gauzes at −20 °C. Before testing, discs were thawed in lukewarm saline water for 30 min.

### Reference disc- and sample preparation

To verify proper functioning of our needle based indenter during *in situ* measurements, we tested a dummy IVD sample in which the NP was replaced with PDMS (Sylgard 184, Dow Corning) with a crosslinker:prepolymer weight ratio of 1:20. The PDMS was mixed, degassed for 30 min, poured into a glass petridish and allowed to cure at room temperature in a flow chamber for 96 hrs. Afterwards, the NP of an IVD was surgically removed by tweezers and a scalpel after drilling a small hole through the bone of the endplate. After NP removal, a cylinder of PDMS was cut from the cured sample and placed firmly into the disc. To fixate the PDMS in all directions the disc was resealed. The remainder of the PDMS in the petridish served as the polymeric reference sample.

### Code availability

The computer code used to generate the results of this study is available on reasonable request from the corresponding author.

### Data availability

All raw and processed data that support the findings of this study are available from the corresponding author upon reasonable request.
