# Publisher Correction: Minimally Invasive Micro-Indentation: mapping tissue mechanics at the tip of an 18G needle

**DOI:** 10.1038/s41598-018-23164-1

**Published:** 2018-03-15

**Authors:** Steven V. Beekmans, Kaj S. Emanuel, Theodoor H. Smit, Davide Iannuzzi

**Affiliations:** 1 Department of Physics and Astronomy and LaserLab Amsterdam, Vrije Universiteit Amsterdam, De Boelelaan 1085, 1081 HV Amsterdam, Netherlands; 2 Department of Orthopaedic Surgery, VU University Medical Center (VUmc), Amsterdam Movement Sciences, De Boelelaan 1117, 1081 HV Amsterdam, Netherlands; 3 Department of Medical Biology and Department of Orthopedic Surgery, Academic Medical Center (AMC), Meiberdreef 9, 1105 AZ Amsterdam, Netherlands

Correction to: *Scientific Reports* 10.1038/s41598-017-10526-4, published online 12 September 2017

This Article contains errors in the Methods section under subheading ‘Dynamic mechanical analysis’.

“Upon contact with the tissue, the feedback-controlled piezoelectric transducer moved the probe forward (thus bending the cantilever) until a predefined value for the applied load (~300) by the cantilever was reached. This load was kept stable for at least 60 s to allow for dissipation of the tissue. Afterwards, the load was oscillated sequentially (amplitude ~10) for a finite number of increasing frequencies (5 periods each), logarithmically spaced between 0.05 Hz (0.5 Hz for the NP measurements) and 10 Hz. During all indentations it was ensured that the indentation depth stayed within the linear viscoelastic regime and that indentation depth was much smaller than the bead radius (the maximum static indentation depth was 40).”

should read:

“Upon contact with the tissue, the feedback-controlled piezoelectric transducer moved the probe forward (thus bending the cantilever) until a predefined value for the applied load (~300µN) by the cantilever was reached. This load was kept stable for at least 60 s to allow for dissipation of the tissue. Afterwards, the load was oscillated sequentially (amplitude ~10µN) for a finite number of increasing frequencies (5 periods each), logarithmically spaced between 0.05 Hz (0.5 Hz for the NP measurements) and 10 Hz. During all indentations it was ensured that the indentation depth stayed within the linear viscoelastic regime and that indentation depth was much smaller than the bead radius (the maximum static indentation depth was 40 µm).”

There are additional errors under the subheading ‘Measurement protocol’.

“After finding contact with the tissue, the probe was retracted for 100 and the dynamic mechanical analysis procedure was started (see section Dynamic mechanical analysis).”

should read:

“After finding contact with the tissue, the probe was retracted for 100 µm and the dynamic mechanical analysis procedure was started (see section Dynamic mechanical analysis).”

